# Spectrum and trigger identification of hemophagocytic lymphohistiocytosis in adults: A single-center analysis of 555 cases

**DOI:** 10.3389/fimmu.2022.970183

**Published:** 2022-08-12

**Authors:** Yi Miao, Jing Zhang, Qingqing Chen, Lingxiao Xing, Tonglu Qiu, Huayuan Zhu, Li Wang, Lei Fan, Wei Xu, Jianyong Li

**Affiliations:** ^1^ Department of Hematology, the First Affiliated Hospital of Nanjing Medical University, Jiangsu Province Hospital, Nanjing, China; ^2^ Key Laboratory of Hematology of Nanjing Medical University, Nanjing, China; ^3^ Pukou Chronic Lymphocytic Leukemia (CLL) Center, Nanjing, China; ^4^ National Clinical Research Center for Hematologic Diseases, the First Affiliated Hospital of Soochow University, Suzhou, China

**Keywords:** hemophagocytic lymphohistiocytosis, lymphoid malignancies, Epstein-Barr virus, aggressive natural killer-cell leukemia, B-cell non-Hodgkin lymphoma

## Abstract

Limited data are available about the underlying causes of hemophagocytic lymphohistiocytosis (HLH) in adults. We collected and analyzed the data of 555 cases of adult HLH. HLH in 242 patients were malignancies-related and lymphoid malignancies (42.0%, 233/555) were the most common causes. Aggressive natural killer-cell leukemia, diffuse large B-cell lymphoma, and extranodal natural killer/T-cell lymphoma, nasal type were the most common specified pathological subtypes. Epstein-Barr virus (EBV) (69.0%, 100/145) was the most common pathogen among the cases of infections-related HLH (26.1%, 145/555). Malignancies-related HLH showed male preponderance, more common splenomegaly, more severe anemia and thrombocytopenia, and significantly elevated soluble CD25. In patients with abnormal lymphoid cells in the bone marrow (BM) and increased EBV DNA copy number, 48.9% (45/92) of them were aggressive natural killer-cell leukemia. In patients with abnormal lymphoid cells in the BM and normal EBV DNA copy number, 66.2% (47/71) of them were B-cell non-Hodgkin lymphoma. In patients with elevated EBV DNA copy number but no abnormal lymphoid cells in the BM, 71.0% (98/138) of these cases were EBV infection. In conclusion, lymphoid malignancy is the most common underlying cause of adult HLH, followed by EBV infection. Based on the BM morphology and EBV load, we developed a diagnostic flow for rapid determination of the triggers for HLH.

## Introduction

Hemophagocytic lymphohistiocytosis (HLH) is an uncommon, devastating disorder that is characterized by an overwhelming inflammatory cytokine storm. Clinically, patients always present with persistent fever, pancytopenia, hepatosplenomegaly, and hyperferritinemia. The hemophagocytosis phenomenon in the bone marrow (BM) could be always observed but is not mandatory for the diagnosis of HLH (1). Traditionally, based on the etiologies, HLH could be classified into primary HLH and secondary HLH (2). Patients with primary HLH are always young children and frequently have genetic defects (3). Secondary HLH, mainly identified in adults, is mostly triggered by underlying secondary causes including malignancies, infections, or autoimmune diseases (4). However, genetic defects may also contribute to the development of adult HLH cases or secondary HLH cases (5).

Although the exact incidence of HLH in adults remains unknown, the number of cases reported in the literature has dramatically increased over the last two decades (6). In contrast to pediatric HLH, the appropriate work-up and treatment for HLH in adults are less well defined. Currently, there is no consensus on the treatment of adults with HLH. In some patients, the HLH-94 or HLH-2004 regimen could be used to control the overwhelming inflammations in the induction treatment of HLH (7, 8), however, the subsequent therapeutic options depend on the underlying causes.

For adults with HLH, it is of vital importance to identify the secondary triggers to select the appropriate treatments. Better description and characterization of the underlying causes for HLH may facilitate the prompt identification of triggers in adult patients in the future. Currently, most data regarding HLH in adults come from small cohorts. Although one recent large multicenter study including 500 adults with HLH was recently published, a detailed description of the underlying causes was not available (9). For instance, for patients with lymphoma, detailed information about the pathological subtypes was not provided (9). In this study, we did a single-center analysis of 555 cases of HLH in adults. We detailed described the spectrum of the underlying causes for these patients and analyzed the clinical and laboratory features of the cases. Furthermore, based on Epstein-Barr virus (EBV) and BM morphology studies, we try to establish a simple diagnostic strategy that may promote the timely identification of triggers in adult patients with HLH.

## Methods

### Patient selection

Patients with HLH aged 18 and older were included in our study. The diagnosis of HLH was established according to the HLH-2004 criteria. Cases of HLH should fulfill 5 or more of following criteria: (1) Body temperature ≥ 38.5°C; (2) Splenomegaly; (3) Cytopenia affecting at least 2 of 3 lineages in the peripheral blood: hemoglobin < 90 g/L, platelets < 100×10^9^/L or neutrophils < 1×10^9^/L; (4) Hypertriglyceridemia and/or hypofibrinogenemia: triacylglycerol ≥ 3 mmol/L, or fibrinogen ≤ 1.5 g/L; (5) Serum ferritin ≥ 500 µg/L; (6) Hemophagocytosis found in BM or spleen or lymph nodes; (7) Soluble CD25 (sCD25) ≥ 2400 U/mL; (8) Natural killer (NK) cell activity is low or absent.

### Data collection

Demographic data including age and gender were collected. Laboratory data for the diagnosis of HLH and copy number of EBV DNA were also collected. We categorized the underlying causes of HLH into four groups: malignancies, infections, rheumatic diseases, and unknown origins. The diagnosis of lymphoid malignancy was made according to the WHO criteria in the majority of cases; however, we made an unspecified diagnosis based on the morphology with or without immunophenotypic evidence in some cases. For diagnosing BM large B cell lymphoma, we reviewed the imaging data to ensure that there was no overt lymphadenopathy or tumor formation to exclude the possibility of diffuse large B-cell lymphoma (DLBCL) with secondary BM involvement.

### Statistical analyses

The D’Agostino-Pearson test was used for examining the normality of continuous variables. If the values passed the normality test and equal variance test, we used Student’s t-test, otherwise, we used the Mann-Whitney test (two groups) or Kruskal-Wallis test (more than two groups). Fisher’s exact test or chi-square test was used to compare categorical variables. Statistical analyses were performed using Prism version 8.2.1 (GraphPad Software). P < 0.05 (2-sided) was considered statistically significant.

## Results

### Baseline characteristics

A total of 555 cases of HLH were identified. The median age at diagnosis of HLH was 51 years old. The male to female ratio was 1.4:1. Almost 97.4% and 86.1% of patients presented with fever and splenomegaly, respectively. Totally, 83.6% of patients showed cytopenia affecting at least two lineages. Hyperferritinemia and elevated sCD25 were detected in 97.5% and 97.7% of patients. Nearly 75.0% of patients showed hypertriglyceridemia and/or hypofibrinogenemia. Hemophagocytosis in BM, spleen or lymph nodes was found in 87.8% of patients. NK cell function was available for 11 patients, and all of them showed decreased NK cell function (**Table 1**).

**Table 1:** Clinical characteristics of 555 cases of hemophagocytic lymphohistiocytosis.

Clinical characteristics of patients	n/N (%)
Age, median (range), years	51 (18-88)
Women	236/555 (42.5)
No. of hemophagocytic lymphohistiocytosis criteria fulfilled	
5	221/555 (39.8)
6	207/555 (37.3)
7	123/555 (22.2)
8	4/555 (0.7)
Fever≥ 38.5°C	535/549 (97.4)
Splenomegaly	458/532 (86.1)
Cytopenia (≥ 2 of 3 lineages)	464/555 (83.6)
Hemoglobin < 90 g/L	353/555 (63.6)
Platelets < 100×10^9^/L	484/555 (87.2)
Neutrophils < 1×10^9^/L	228/555 (41.1)
Hypertriglyceridemia and/or hypofibrinogenemia	415/553 (75.0)
Triacylglycerol ≥ 3 mmol/L	255/553 (46.1)
Fibrinogen ≤ 1.5 g/L	318/552 (57.6)
Soluble CD25 ≥ 2400 U/mL	335/343 (97.7)
Soluble CD25 >10000 U/mL	267/343 (77.8)
Ferritin ≥ 500 μg/L	539/553 (97.5)
Hemophagocytosis	484/551 (87.8)

### Analysis of etiologies

**Figure 1:**
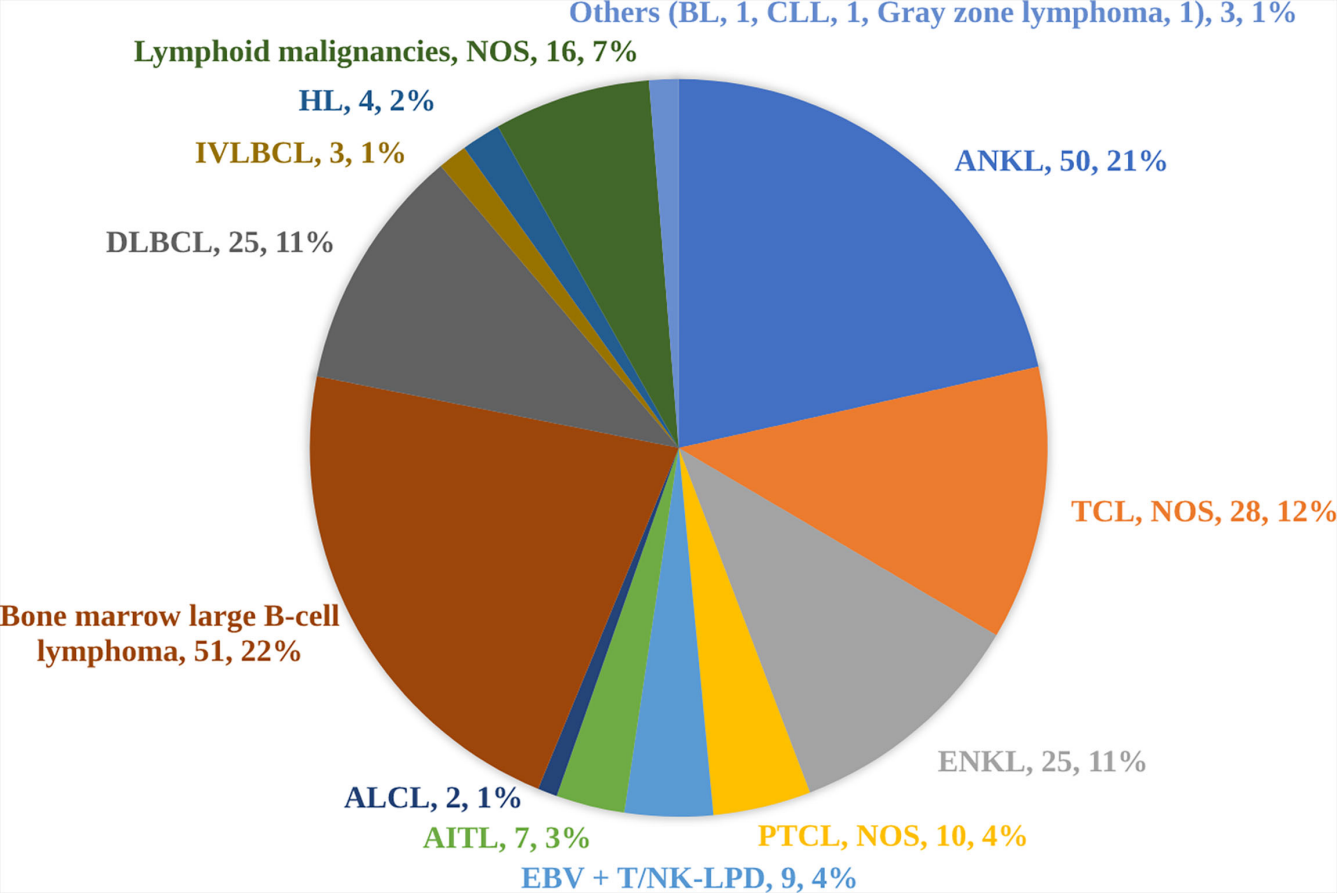
Subtypes of 233 cases of lymphoid malignancies triggering hemophagocytic lymphohistiocytosis. As bone marrow large B-cell lymphoma is not present in the WHO classification, it was therefore not a specified subtype in our study. ALCL, anaplastic large-cell lymphoma; AITL, angioimmunoblastic T-cell lymphoma; ANKL, aggressive natural killer-cell leukemia; BL, Burkitt lymphoma; CLL, chronic lymphocytic leukemia; DLBCL, diffuse large B-cell lymphoma; EBV+ T/NK-LPD, Epstein-Barr virus-associated T/natural killer-cell lymphoproliferative disorders; ENKL, extranodal natural killer/T-cell lymphoma, nasal type; HL, Hodgkin lymphoma; IVLBCL, intravascular large B-cell lymphoma; NOS, not otherwise specified; PTCL, peripheral T-cell lymphoma; TCL, T-cell lymphoma.

Underlying causes were identified in 76.2% (423/555) of cases. HLH in 242 patients was tumor-related and among these patients, 233 patients were diagnosed with lymphoid malignancies. Among 233 patients with lymphoid malignancies, 131 patients had T/NK-cell malignancies, 82 patients had B-cell non-Hodgkin lymphoma (B-NHL), and four patients had Hodgkin lymphoma. Among T/NK-cell malignancies, aggressive NK-cell leukemia (ANKL) (n=50) and extranodal NK/T-cell lymphoma, nasal type (ENKL) (n=25) were the most common specified subtypes. Among B-NHL, there were twenty-five cases of DLBCL (containing 5 EBV-positive DLBCL confirmed by EBER staining) and 51 cases were B-cell lymphoma predominately affecting the BM, which we named BM large B-cell lymphoma. As BM large B-cell lymphoma is not present in the WHO classification, it was therefore not a specified subtype in our study. Other subtypes were summarized in **Figure 1**. A total of 145 cases of HLH were attributed to infections, among which EBV (69.0%, 100/145) was the most common pathogen. Nine cases of HLH were novel Bunyavirus-related. Additionally, HLH associated with histiocytic necrotizing lymphadenitis was diagnosed in 5 patients. There were 36 cases of HLH associated with rheumatic diseases. In these cases, systemic lupus erythematosus (12/36) and adult-onset Still’s disease (6/36) are two main underlying causes.

### Differences in clinical and laboratory features among groups based on etiologies

**Figure 2:**
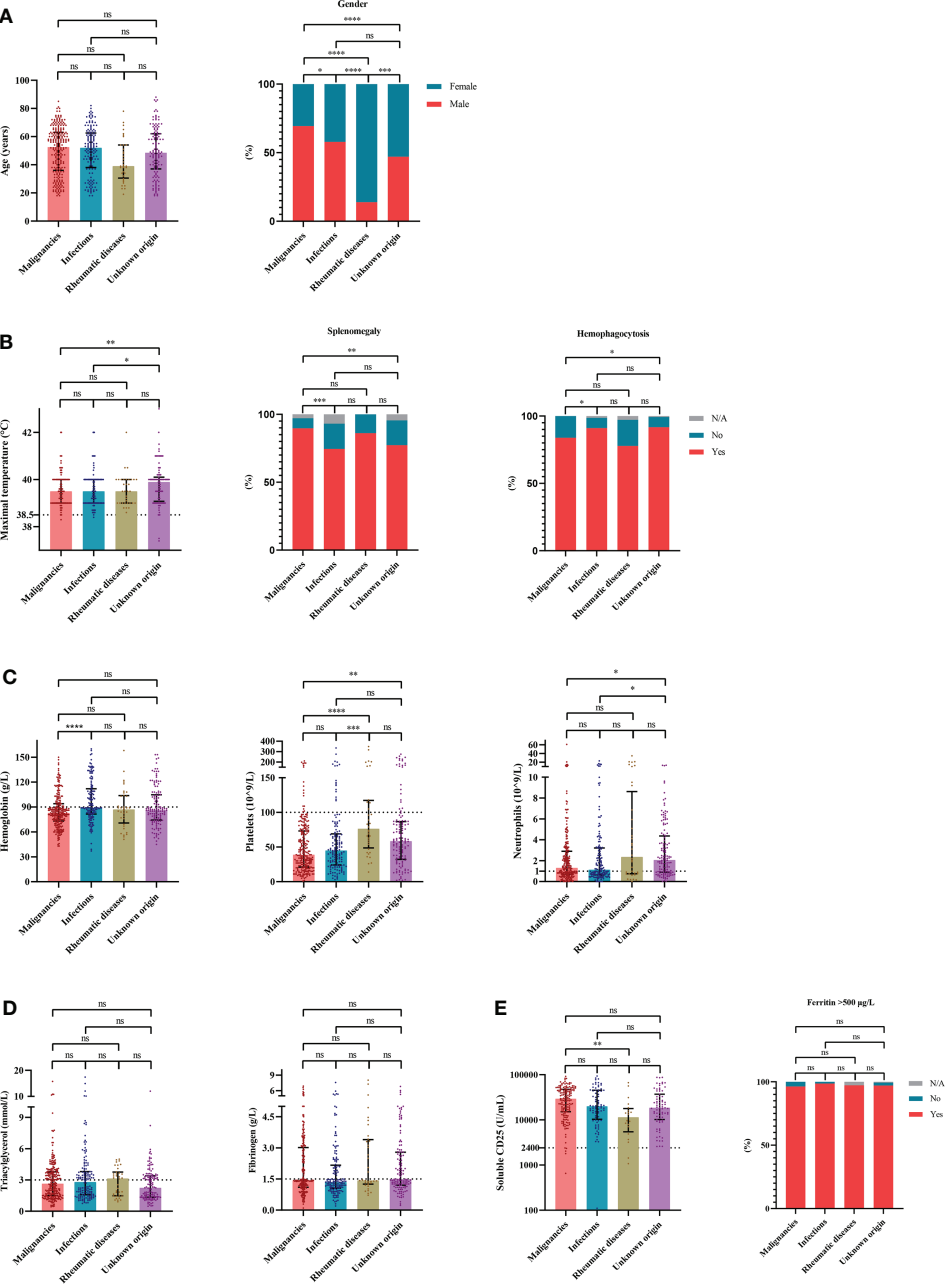
The distribution of clinical and laboratory features reveals differences among cases of hemophagocytic lymphohistiocytosis based on etiologies. **(A)** Distribution of the demographic data. **(B)** Distribution of the clinical manifestation and histopathological findings. **(C)** Distribution of the blood lineages. **(D, E)** Distribution of other laboratory findings. *P < 0.05, **P < 0.01, ***P < 0.001, ****P < 0.0001. ns, not significant.

**Figure 3:**
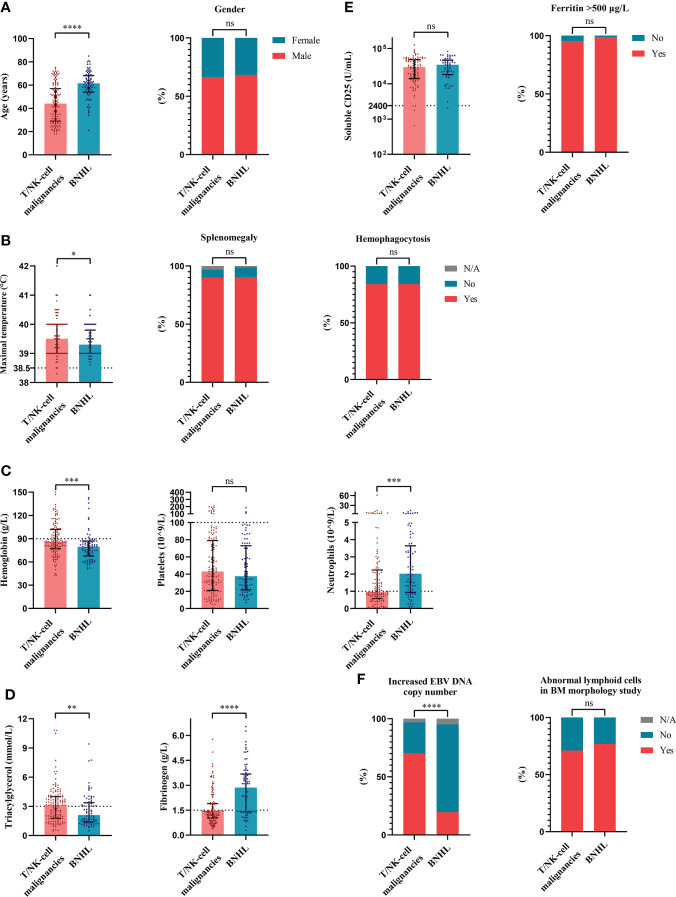
The distribution of clinical and laboratory features reveals differences between cases of hemophagocytic lymphohistiocytosis triggered by T/NK-cell malignancies and B-NHL. **(A)** Distribution of the demographic data. **(B)** Distribution of the clinical manifestation and histopathological findings. **(C)** Distribution of the blood lineages. **(D, E)** Distribution of other laboratory findings. **(F)** Distribution of the EBV infection and BM morphology findings. *P < 0.05, **P < 0.01, ***P < 0.001, ****P < 0.0001. BM, bone marrow; B-NHL, B-cell non-Hodgkin lymphoma; EBV, Epstein-Barr virus; ns, not significant. T/NK-cell malignancies, T/natural killer-cell malignancies.

To evaluate the characteristics of HLH triggered by different underlying causes, we compared variables of interest among the four groups based on etiologies. The cases of HLH associated with malignancies showed male preponderance compared with other groups (**Figure 2A**). More common splenomegaly, more severe anemia, however, less common hemophagocytosis were seen in the cases of malignancies-associated HLH than the cases of infections-associated HLH (**Figure 2**). Compared with the cases of HLH triggered by rheumatic diseases, the cases of HLH associated with malignancies showed significantly elevated sCD25 and severe thrombocytopenia (**Figure 2**). Additionally, we compared the clinical and laboratory features between cases of HLH triggered by T/NK-cell malignancies and B-NHL (**Figure 3**). We found that cases of T/NK-cell malignancies presented with younger age, higher maximal temperature, lower counts of neutrophils, more severe hypertriglyceridemia and hypofibrinogenemia, and more frequent EBV infection. By contrast, cases of B-NHL showed more severe anemia (**Figure 3**).

### Active EBV infections in patients with lymphoid malignancies-associated HLH

**Figure 4:**
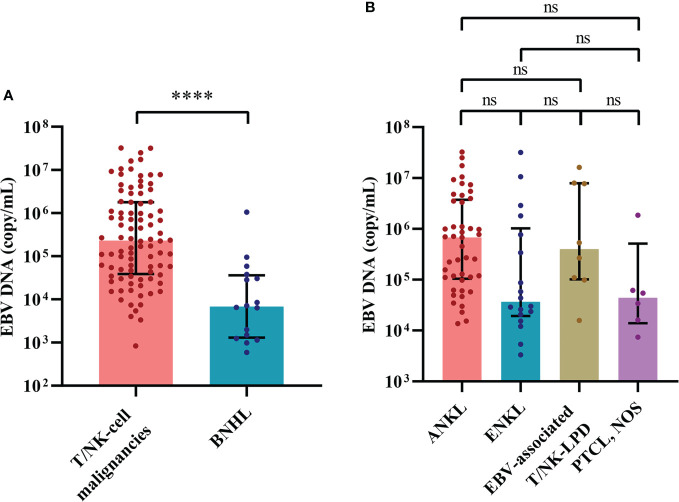
The distribution of EBV load reveals differences between cases of hemophagocytic lymphohistiocytosis triggered by T/NK-cell malignancies and B-NHL. **(A)** Distribution of the EBV load between cases of T/NK-cell malignancies-associated and B-NHL-associated hemophagocytic lymphohistiocytosis. **(B)** Distribution of the EBV load among the commonly specified subtypes of T/NK-cell malignancies. ****P < 0.0001. ANKL, aggressive natural killer-cell leukemia; B-NHL, B-cell non-Hodgkin lymphoma; EBV, Epstein-Barr virus; EBV-associated T/NK-LPD, Epstein-Barr virus-associated T/natural killer-cell lymphoproliferative disorders; ENKL, extranodal natural killer/T-cell lymphoma, nasal type; ns, not significant. PTCL, NOS, peripheral T-cell lymphoma, not otherwise specified; T/NK-cell malignancies, T/natural killer-cell malignancies.

Among 518 patients who were examined for the whole-blood EBV DNA copy number, 232 patients showed elevation of EBV DNA copy numbers, which suggested active EBV infection. A total of 222 cases of lymphoid malignancy-associated HLH were tested for the whole-blood EBV DNA copy number and 122 cases showed elevation of EBV DNA copy numbers. We categorized these cases with lymphoid malignancy into the EBV-positive (EBV+) group and the EBV-negative (EBV-) group. We found that the EBV+ group was enriched for T/NK-cell neoplasms (75.4%, 92/122). Sixty-two percent of cases (62/100) in the EBV- group were B-NHL. In EBV+ cases (**Figure 4**), the cases of T/NK-cell malignancies-associated HLH showed significantly elevated EBV DNA copy numbers compared with the cases of B-NHL-associated HLH (**Figure 4A**). When considering the commonly specified subtypes of T/NK-cell malignancies, ANKL seemed to have higher EBV DNA copy numbers, however, no significant difference was observed (**Figure 4B**).

### Combining BM morphology study and EBV status to determine the triggers for HLH

**Figure 5:**
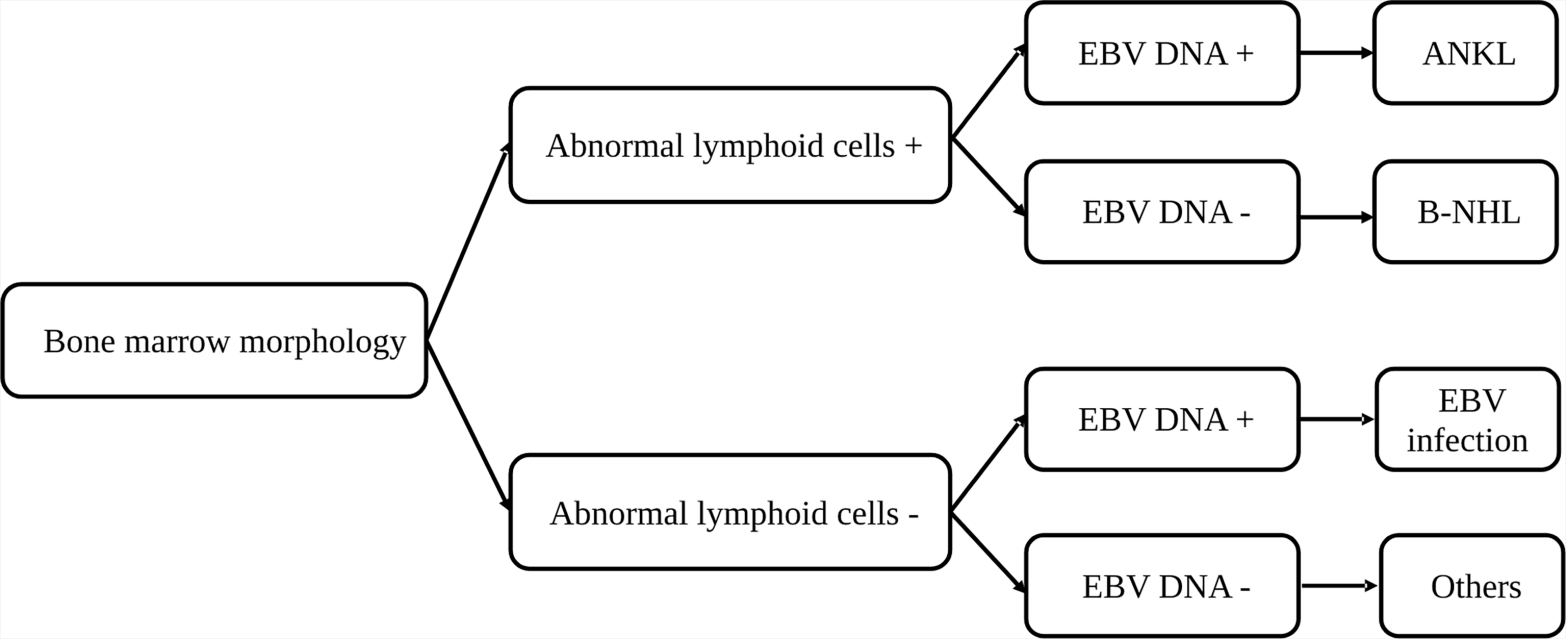
A diagnostic flow for rapid determination of the triggers for hemophagocytic lymphohistiocytosis based on the bone marrow morphology and EBV copy number. ANKL, aggressive natural killer-cell leukemia; B-NHL, B-cell non-Hodgkin lymphoma; EBV, Epstein-Barr virus.

As BM smear and EBV DNA copy number evaluation are essential studies in the determination of secondary causes for HLH, we hypothesized that a combination of these two examinations may help in finding the triggers for adult HLH and developed a diagnostic flow for rapid determination (**Figure 5**). We divided patients with BM smear and EBV DNA copy number into four groups (**Table 2**): patients with abnormal lymphoid cells and increased EBV DNA copy number (BM+EBV+), patients with abnormal lymphoid cells and normal EBV DNA copy number (BM+EBV-), patients with increased EBV DNA copy number but without abnormal lymphoid cells (BM-EBV+), and patients without abnormal lymphoid cells and with normal EBV DNA copy number. We found that in BM+EBV+ patients, 48.9% (45/92) of these cases were ANKL. And for BM+EBV- cases, 66.2% (47/71) of them are B-NHL, with BM large B cell lymphoma accounting for the majority. In BM-EBV+ patients, 71.0% (98/138) of these cases were classified as EBV infection-related HLH.

**Table 2:** Underlying disease in patients with different bone marrow morphology and Epstein-Barr virus load.

Disease	n	Percent	Disease	n	Percent
**BM+EBV+**	92		**BM+EBV-**	71	
Aggressive natural killer-cell leukemia	45	48.9	BM large B-cell lymphoma	37	52.1
Lymphoma, not otherwise specified	11	12.0	T-cell lymphoma, not otherwise specified	14	19.7
BM large B-cell lymphoma	8	8.7	Diffuse large B-cell lymphoma	5	7.0
Extranodal natural killer/T-cell lymphoma, nasal type	7	7.6	Aggressive natural killer-cell leukemia	4	5.6
T-cell lymphoma, not otherwise specified	6	6.5	Others*	11	15.5
Epstein-Barr virus-associated T/natural killer-cell lymphoproliferative disorders	5	5.4			
Angioimmunoblastic T-cell lymphoma	4	4.4	**BM-EBV-**	214	
Others*	6	6.5	Unknown origin	114	53.3
			Diffuse large B-cell lymphoma	12	5.6
**BM-EBV+**	138		Bacterial infection	9	4.2
EBV infection	98	71.0	Novel Bunyavirus infection	8	3.7
Extranodal natural killer/T-cell lymphoma, nasal type	11	8.0	Other viral infection	7	3.3
Peripheral T-cell lymphoma, not otherwise specified	5	3.6	Systemic lupus erythematosus	7	3.3
Other viral infection	4	2.9	T-cell lymphoma, not otherwise specified	5	2.3
Others*	20	14.5	Histiocytic necrotizing lymphadenitis	5	2.3
			Adult-onset Still’s disease	5	2.3
			Sjögren’s syndrome	5	2.3
			Peripheral T-cell lymphoma, not otherwise specified	4	1.9
			Others*	33	15.4

BM+, abnormal lymphoid cells in bone marrow smear; BM-, normal lymphoid cells in bone marrow smear; EBV+, increased EBV DNA copy number; EBV-, normal EBV DNA copy number. *Specific subtypes with case number less than 4 were not specified in this table.

## Discussion

In this study, by using a large cohort of 555 adult patients with HLH, we analyzed the spectrum of adult HLH. We defined the underlying causes of HLH and found the most common secondary cause of HLH was malignancy, which was followed by infection. For malignancy-related HLH, most cases were related to lymphoid malignancies, of which the most common specified pathological subtype was ANKL. And for infection-related HLH, the EBV virus was the most common pathogen. Furthermore, based on the BM morphology study and EBV copy number evaluation, we developed a diagnostic flow that could be used for rapid determination of the triggers for HLH in at least a proportion of patients.

Consistent with other studies (10–13), lymphoid malignancy was the most common secondary cause of adult HLH. Regarding subtypes, ANKL and ENKL were much more common than those reported in cohorts from western countries (13, 14). Similarly, according to a study involving 264 HLH cases from Korea, the most common secondary cause was lymphoid malignancy, among which ENKL was the most common subtype (15). And this could be due to the fact that ENKL and ANKL are much more prevalent in Asian countries than in western countries (16). And patients with ANKL or ENKL frequently present with HLH. ANKL is a relatively rare disease. According to the study by Li et al. and the study by Song et al, approximately 60% of patients with ANKL developed HLH (17, 18). ENKL is more common than ANKL. And the cumulative risk of developing HLH ranges from 7.1%-13.3% in patients with ENKL (19–21). Therefore, the prevalence of ANKL or ENKL-related HLH in our cohort may be attributed to the high incidence of ANKL/ENKL in the Asian population. Other common subtypes of lymphomas associated with HLH in our study included DLBCL and BM large B-cell lymphoma. HLH related to DLBCL has been reported in several studies. And in some studies, DLBCL is the most common subtype of lymphoma that is related to HLH (22, 23). In our study, of 25 DLBCL cases, 5 cases were EBV-positive DLBCL. According to a recently published study, 14% of patients with EBV-positive DLBCL presented with HLH, suggesting EBV-positive DLBCL is also a common subtype that is associated with HLH (24). Interestingly, we have 51 cases of BM large B-cell lymphoma. These cases could only be classified as BM large B-cell lymphoma due to the lack of non-BM biopsy tissues. Primary bone marrow lymphoma has been reported (25). In a study of 30 cases of primary bone marrow lymphoma, in which the most common pathological subtype was large B cell lymphoma, HLH was an initial presentation in 67% (20/30) of these cases. The high co-occurrence of HLH and primary BM lymphoma suggested these BM large B-cell lymphoma could be classified as primary BM large B-cell lymphoma (25). And Iioka et al. (26) reported 10 cases of diffuse large B-cell lymphoma confined to the BM, spleen, and liver. In these 10 cases, fever, splenomegaly, cytopenia, hyperferritinemia, elevated sCD25, and hemophagocytosis were frequent, indicating the presence of HLH. This study emphasized the linkage between primary BM large B cell lymphoma and HLH. However, for these 51 cases, intravascular large B cell lymphoma (IVLBCL) could not be excluded. Patients with IVLBCL Asian variant frequently present with HLH and primarily BM lymphoma infiltration (27). Although for these 51 cases, IVLBCL was not diagnosed, it should be noted that BM biopsy alone could only identify a minority of IVLBCL cases (28). For those with a massive nodular or interstitial infiltration pattern in the BM, non-BM biopsy, especially random skin biopsy is essential for the diagnosis of IVLBCL (29). There are a few possible mechanisms for lymphoma-associated HLH. First, persistent antigen stimulation and hypersecretion of cytokines by tumor cells may contribute to the development of lymphoma-associated HLH (30). Second, germline mutations may predispose individuals to both lymphoma and HLH, for instance, germline HAVCR2 mutations promote the development of HLH and subcutaneous panniculitis-like T cell lymphoma (31). Third, for EBV-related lymphoma, the high load of EBV is also a trigger for HLH.

In our study, EBV was the most common pathogen among cases of HLH triggered by infection, accounting for 18% (100/555) of the entire cohort. Several studies from China and South Korea have revealed EBV infection as the most common or secondary cause of HLH in adults (9, 11, 32), which can be attributed to the prevalence of EBV in Asia. Additionally, there were nine cases of HLH triggered by the novel Bunyavirus in our cohort, which was absent in an epidemiological investigation of HLH in China (9). It might be explained by the geographic variations in the novel Bunyavirus as it is endemic in central and eastern China (33), whereas most cases were from northern China in the above study.

When comparing the clinical and laboratory features among groups based on etiologies, we found that the cases of HLH associated with malignancies were characterized by male predominance and splenomegaly, as well as more severe anemia and thrombocytopenia. It was mainly due to the large proportion of lymphomas in malignancies. Lymphomas may involve the spleen, thereby contributing to splenomegaly. And lymphomas also frequently invade the bone marrow and aggravate cytopenia. Notably, the median sCD25 levels of the four groups were more than 10000 U/mL and were significantly elevated in malignancies-related HLH compared with the cases of HLH triggered by rheumatic diseases. Consistent results were reported by Hayden et al. (34), who demonstrate that a level of 2400 U/mL or less helped rule out HLH and more than 10000 U/mL helped diagnose HLH. These results suggest that sCD25 is of vital diagnostic value for HLH and its triggers.

Timely recognition and treatment of HLH are essential since it was reported that early etoposide administration was correlated to survival (35). A minimal parameter set using 2 major criteria and 3 minor criteria was applied for early diagnosis of HLH, mainly in pediatric cases. However, the underlying causes of adult HLH are more complicated and lymphoma-related HLH, especially those related to T/NK-cell malignancies, were reported to have a poorer prognosis compared with other etiologies (22, 35–38). Therefore, early identification of lymphoma from patients with HLH was of vital importance. Thus, we developed a diagnostic flow based on BM smear and EBV DNA load, which are clinically feasible examinations. It might help to discriminate cases of HLH triggered by T/NK- and B-cell malignancies, as well as EBV infection. However, this diagnostic flow has limitations. For example, abnormal lymphoid cells in BM and the concurrently increased EBV-DNA copies may also be linked to EBV-positive DLBCL and other subtypes, although ANKL was the most common subtype. Therefore, in this circumstance, essential differential diagnosis should be made.

## Conclusions

By analyzing a cohort of 555 cases with HLH, we systematically investigated the spectrum and triggers of adult HLH in the Chinese population. Our study demonstrates that lymphoid malignancy is the most common underlying cause of adult HLH, followed by EBV infection. And among lymphoid malignancies, ANKL, ENKL, and DLBCL were the most common specified subtypes. Based on the bone marrow morphology and EBV load, we developed a diagnostic flow for rapid determination of the triggers for HLH.

## Data availability statement

The raw data supporting the conclusions of this article will be made available by the authors, without undue reservation.

## Ethics statement

The studies involving human participants were reviewed and approved by the Ethics Committee of Jiangsu Province Hospital. The patients/participants provided their written informed consent to participate in this study.

## Author contributions

YM, JZ, and QC: Conceptualization, methodology, data curation, formal analysis, writing-original draft, and visualization. LX and TQ: Data curation, formal analysis, and visualization. HZ, LW, and LF: Conceptualization, writing-review & editing, and supervision. WX and JL: Conceptualization, methodology, funding acquisition, supervision, writing-review & editing. All authors contributed to the article and approved the submitted version.

## Funding

National Natural Science Foundation of China (Grant No. 82100207 and Grant No. 81720108002), Natural Science Foundation of Jiangsu Province (Grant No. BK20210962), and Translational Research Grant of NCRCH (2020ZKZB01).

## Conflict of interest

The authors declare that the research was conducted in the absence of any commercial or financial relationships that could be construed as a potential conflict of interest.

## Publisher’s note

All claims expressed in this article are solely those of the authors and do not necessarily represent those of their affiliated organizations, or those of the publisher, the editors and the reviewers. Any product that may be evaluated in this article, or claim that may be made by its manufacturer, is not guaranteed or endorsed by the publisher.
